# Is a Highly Linear Relationship Between the Dose of Quercetin and the Pharmacological Effect Possible? — A Comment on Liu, *et al*. Evaluation of Antioxidant and Immunity Activities of Quercetin in Isoproterenol-Treated Rats. *Molecules* 2012*, 17,* 4281–4291

**DOI:** 10.3390/molecules19079606

**Published:** 2014-07-07

**Authors:** Přemysl Mladěnka, Radomír Hrdina, Tomáš Filipský, Michal Říha, Vladimir Palicka

**Affiliations:** 1 Department of Pharmacology and Toxicology, Charles University in Prague, Faculty of Pharmacy in Hradec Králové, Heyrovského 1203, 500 05 Hradec Králové, Czech Republic; 2 Charles University in Prague, Faculty of Medicine in Hradec Králové, Šimkova 870, 500 38 Hradec Králové, Czech Republic; 3 University Hospital Hradec Králové, Sokolská 581, 500 05 Hradec Králové, Czech Republic

We wish to offer some comments on the article by H. Liu *et al*. entitled “Evaluation of antioxidant and immunity activities of quercetin in isoproterenol-treated rats”, published in *Molecules* in 2012 [1]. First of all, there are several important points which are not adequately explained in the article and require clarification by the authors:

- No information is provided on how the quercetin was dissolved and administered.
- There are no illustrative ECG measurements nor any comment on how the ECG signals were measured, this necessitates comments due to the measured T wave depression data.
- Heart index is not specified and since this is not a very common parameter, how was it calculated?
- The statistical test used by the authors in the comparison of data is not mentioned.
- Did all isoprenaline-treated animals survive the experiment?

Secondly, the design of the study and thus the possible outcome may be misleading since it appears no control groups which only received quercetin were included in the study. This may markedly influence the study results, e.g., it is possible that oral administration of quercetin might result in gradual increases in antioxidant levels and decreases in pro-inflammatory factors, and this may counterbalance the effect of isoprenaline leading to the opposite processes. Therefore it is not clear if quercetin had acute effects by blocking the activity of isoprenaline or if it chronically decreases oxidative stress.

The third and the most important issue is an unnaturally precise linear relationship reported between the dose of quercetin and its pharmacological effects. We have plotted all measured data from the tables of the discussed article in graphs (Figure 1) with the exception of IL-10, where no protection was found.

**Figure 1:**
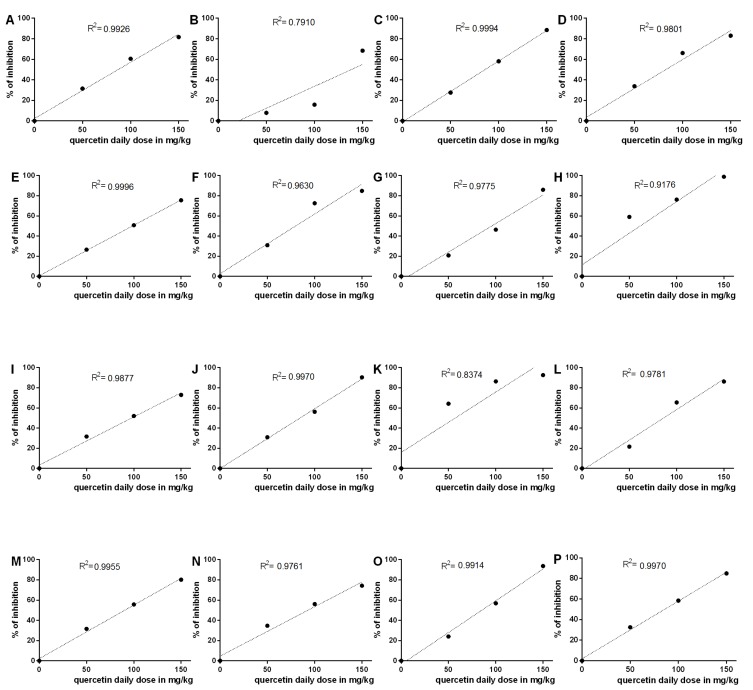

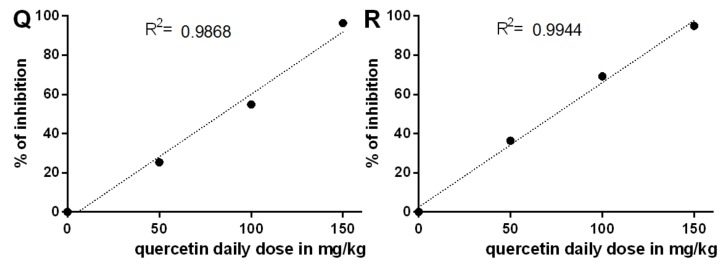
Linear relationship between the daily dose of quercetin and the percentage of inhibition of isoprenaline effect. **A**: T wave-amplitude, **B**: heart rate, **C**: AST, **D**: CK-MB, **E**: LDH, **F**: TNF α, **G**: NO, **H**: nitric oxide synthase, **I**: IL-1, **J**: IL-8, **K**: Na^+^/K^+^-ATPase, **L**: Ca^2+^/Mg^2+^-ATPase, **M**: myeloperoxidase, **N**: TBARS, **O**: glutathione, **P**: superoxide dismutase, **Q**: catalase and **R**: glutathione peroxidase. Coefficients of linear regression (R^2^) were calculated using GraphPad software version 6.0 (San Diego, CA, U.S.A.).

The data were thus elaborated in a way that enabled comparison among the different measured parameters. In short, control values were considered 100% (healthy) and isoprenaline animals were considered 0% (pathological state), and the effect of quercetin was expressed as per cent inhibition of the isoprenaline effect. The blockade of isoprenaline activity was always dose dependent and moreover in eight out of 18 cases (44%) the linear regression coefficient was higher than 0.99 and in a total of 14 cases out of 18 (78%) the mentioned coefficient was higher than 0.975. It is not very probable to achieve such value in so many measured parameters. These results do not resemble *in vivo* experiments, but rather calibration curves. Moreover, according to literature precedents a dose dependent and clearly linear normalization of T wave amplitude is highly improbable. Notwithstanding it is not clear which ECG lead was recorded and the T wave amplitude units were not defined, their values do not correspond to the normal rat ECG. In a respected paper of Beinfeld and Lehr the following mean values of normal T wave amplitude in millivolts were described: lead I = 0.070, lead II = 0.145, lead III = 0.130, aVF = 0.150, aVL= −0.045 and aVR = −0.105 [2]. The mean value given in the article of Liu *et al.* of 2.68 (no units given) clearly does not fit in the above-mentioned range of normal amplitude T wave values. Additionally, it is well known that pharmacokinetics of oral quercetin are nonlinear [3,4,5,6] and thus a strictly linear relationship between the dose and the effect can be excluded with a high probability. We thus appeal to authors to promptly clarify the mentioned discrepancies.

## References

[1] Liu H., Zhang L., Lu S. (2012). Evaluation of antioxidant and immunity activities of quercetin in isoproterenol-treated rats. Molecules.

[2] Beinfield W.H., Lehr D. (1968). QRS-T variations in the rat electrocardiogram. Am. J. Physiol..

[3] Cialdella-Kam L., Nieman D.C., Sha W., Meaney M.P., Knab A.M., Shanely R.A. (2012). Dose-response to 3 months of quercetin-containing supplements on metabolite and quercetin conjugate profile in adults. Br. J. Nutr..

[4] Ader P., Wessmann A., Wolffram S. (2000). Bioavailability and metabolism of the flavonol quercetin in the pig. Free Radic. Biol. Med..

[5] De Boer V.C., Dihal A.A., van der Woude H., Arts I.C., Wolffram S., Alink G.M., Rietjens I.M., Keijer J., Hollman P.C. (2005). Tissue distribution of quercetin in rats and pigs. J. Nutr..

[6] Egert S., Wolffram S., Bosy-Westphal A., Boesch-Saadatmandi C., Wagner A.E., Frank J., Rimbach G., Mueller M.J. (2008). Daily quercetin supplementation dose-dependently increases plasma quercetin concentrations in healthy humans. J. Nutr..

